# Modeling Oil Recovery for Mixed Macro- and Micro-Pore Carbonate Grainstones

**DOI:** 10.1038/s41598-017-09507-4

**Published:** 2017-08-29

**Authors:** Ye Xu, Qiuzi Li, Hubert E. King

**Affiliations:** Corporate Strategic Research, ExxonMobil Research and Engineering Co, Annandale, NJ 08801 USA

## Abstract

In general, modeling oil-recovery is a challenging problem involving detailed fluid flow calculations with required structural details that challenge current experimental resolution. Recent laboratory experiments on mixed micro- and macro-pore suggest that there is a systematic relationship between remaining oil saturation (ROS) and the fraction of micro-pores. Working with experimental measurements of the pores obtained from X-ray tomography and mercury intrusion capillary pressure porosimetry, we define a digital rock model exemplifying the key structural elements of these carbonate grainstones. We then test two fluid-flow models: invasion percolation model and effective medium model. Although invasion percolation identifies the important impact of macro-pore percolation on permeability, it does not describe the dependence of ROS on micro-pore percentage. We thus modified the effective medium model by introducing a single-parameter descriptor, r_eff_. Oil from pores r ≥ r_eff_ is fully removed, while for the remaining pores with r < r_eff_, their contribution is scaled by (r/r_eff_)^2^. Applying this straightforward physics to pore size distributions for the mixed-pore grainstones reproduces the experimental ROS dependence.

## Introduction

As is well recognized, oil recovery from a rock having a heterogeneous pore size distribution is typically poor when compared to that from one with a homogeneous pore size distribution^1^. Conceptually this observation fits our picture of how an immiscible sweep fluid, such as water, will be diverted into connected pathways of larger pores (higher permeability) characteristic of the heterogeneous pore size distribution. The result is significant bypass of oil. It would be desirable to understand the controls on this geometric-controlled sweep efficiency as it can be anticipated to be important in the many carbonates that contain micro-pores.

Heterogeneity of pore size in carbonates is well known. An important class of such rocks exhibit abundant micro-pores in the same rock where macro-pores are present^2–7^. The microporosity of such reservoir rocks has been an area of extensive study for many years. Recent work has summarized findings from that literature and complemented that with a substantial data set taken over a broad range of Phanerozoic age carbonates^8–10^. This work has identified key structural characteristics and shown their impacts on permeability and recovery factors for water and gas floods. One key finding is that limestone microporosity is hosted in a matrix consisting of low-magnesium calcite micro-crystals with diameters of 0.5 to 9 µm^9^. These crystals are the result of diagenesis, i.e. dissolution and recrystallization. Recently Hasiuk *et al*.^10^ presented a large geochemical dataset that is consistent with simple burial diagenesis. Important for the present study is that the flow characteristics are directly related to the textural characteristics and crystal size. Kaczmarek *et al*.^9^ identified three petrophysical types (I, II, III) consisting of progressively lower porosity, reduced crystal sizes and smaller pore-throat radii. Utilizing data from microporosity-dominated rocks, they find a systematic porosity-permeability trend, across the entire range, going from higher to lower permeability for Types I to III. Therefore for a purely microporous carbonate rocks, the flow characteristics are determined by the micro-pore size. We make use of this fact in our analysis of mixed porosity.

Focusing on Type I microporous carbonates, Fullmer *et al*.^8^ studied the oil recovery characteristics when macro-pores are also present. Utilizing a typical petrographic microscope examination of thin sections, they segment the pore space, dividing the sample porosity into micro and macro components. The macro-porosity cut-off is ~10 μm diameter. Supplemented by a bulk determination of porosity (i.e. helium porosimetry), microporosity is then calculated from the difference. This is Total Pore System (TPS) analysis ^11^. Fullmer *et al*.^8^ noted several key features in the Type I carbonates. First, as microporosity increases, oil recovery increases, (i.e., lower remaining oil saturation, ROS, determined at 1/99 oil/water flow for steady-state relative permeability). As the microporosity percentage approaches 100%, a minimum ROS value is observed. This relationship is shown in Fullmer *et al*.^8^. Figure 16 (the experimental data are reproduced here in Section: Effective Medium Model). In another measurement, the minimum oil saturation, determined by primary imbibition measurements via water-oil centrifugal capillary pressure experiments (Fullmer *et al*.^8^. Figure 14B), also decreases as microporosity increases. A third key observation is that overall sample permeability shows a two decade increase for microporosity <80% percent (Fullmer *et al*.^8^ Figure 10). Fullmer *et al*.^8^ distinguish between regimes of micro-pore and mixed-pore dominated flow at that value.

Grainstones are the focus in the case study by Fullmer *et al*.^8^, and here, we consider their geometry to gain insight into the possible geometric controls on flow. A wide range of carbonate grain types can undergo diagenetic alteration to microporosity^9^. As there are two inherent types of pores, inter-particle and intra-particle, such carbonate rocks can be expected to exhibit a mixed pore size. Following deposition, there are primary interparticle macro-pores. These are altered by diagenetic processes. For example, burial reduces inter-grain porosity due to mechanical and chemical compaction^12^. In general, inter-particle porosity will decrease with diagenesis. On the other hand, diagenesis of the grains themselves drives transformations that lead to micro-pores forming within the grain bodies^9^. For example, see the study by Fabricius^13^ of burial diagenesis for chalk sediments. As this proceeds the intra-particle pores become more interconnected and homogeneous in size, dominated by inter-crystalline space between micro-crystals. An example of the resulting texture is shown in Figure 4 of Fullmer *et al*.^8^. Although mineralogical and textural characteristics of the original grains can influence microporosity formation, the grainstones under study exhibit porosities for grains that produce about 20% microporosity, typical of Type I.

**Figure 1:**
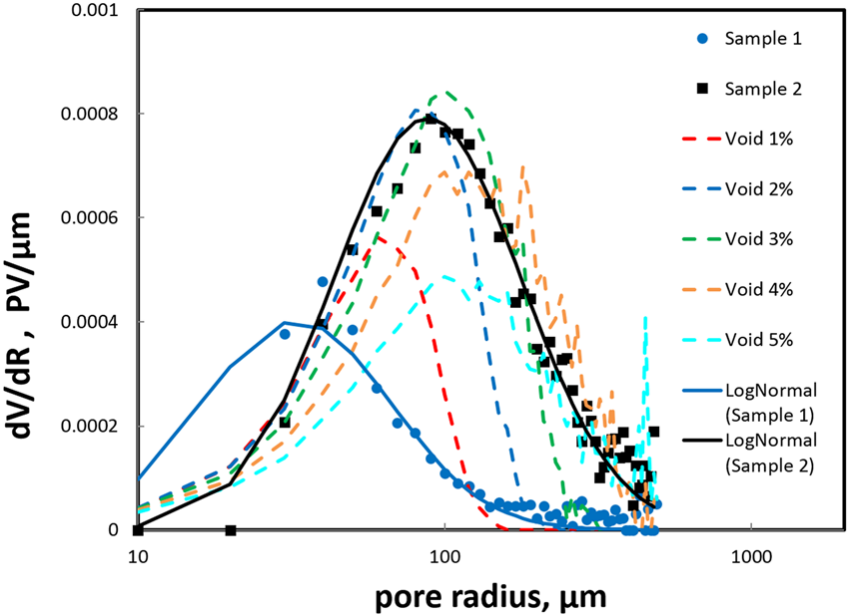
Comparison of macro-pore size distribution for experimentally-determined values (Sample 1 and 2) and those for overlapping-sphere digital rock. Void content indicates sample volume fraction of macro-pores. We avoid the contribution of large, sample-spanning clusters by restricting analysis to samples below void percolation. Solid lines are log-normal fits to the experimental data (see text).

These diagenetically driven variations will alter the pore geometry in a systematic manner. Here, we will address the question of how these geometric changes will affect oil recovery. Because pressure capillary data suggest there is no significant change in wettability^14^, geometric changes most likely drive the recovery factor changes. Consideration of a simple grainstone model allows us to create an ensemble of possible geometric variations. Working within those bounds, we can then examine how oil recovery factor is affected by the geometry of the pore system. Such a study is valuable because the acquisition of experimental data is time consuming. In addition, with a generalized numerical model we can rapidly identify favorable geometries for high reservoir quality, recognizing high quality zones using geometric descriptors alone. Our aim is to identify the key structural parameters and to determine the sensitivity of ROS to variations in this geometry.

In the present work, we first utilize X-ray tomography to analyze the pore size distribution for two carbonate samples, for which ROS has been previously determined. Then, guided by these observations, we generalize the results through creation of a digital rock model that encompasses the key structural information for mixed porosity grainstones, i.e. inter-grain macro-pores and inter-grain micro-pores. We then explore how changing the proportion of each pore type affects predicted ROS. Guided by void percolation modelling^15, 16^, we anticipated macro-pore percentage would have significant impact on flow. Therefore, the first model uses straightforward invasion percolation of the 3D digital rock, exploring how inter-grain void percolation affects swept volume. Finding agreement with an increase in permeability at microporosity = 80%, this model was inadequate to model ROS. Working from an assumption that there is a critical pore size, r_eff_, above which all oil is produced, the second model utilizes an effective medium theory. This single parameter r_eff_, determined uniquely from the pore size distribution, along with an assumption of flow controlled by (r/r_eff_)^2^, gives a model that accurately describes the experimental ROS.

## Experimental Procedures and Results

### Sample Selection

Samples came from the Fullmer *et al*.^8^ case study on rock cores from a large Cretaceous offshore oil reservoir. This study was designed to produce high quality flow data (Special Core Analysis SCAL) aligned with structural data defining the rock types^14^. Recognizing the heterogeneity of carbonate rocks, the study developed a unique plugging and companion rock sampling strategy to insure consistency within a given sample category. It is well known that the rock’s wettability will affect flow properties. Carbonate studies are particularly challenged for these rocks, as there is no recognized manner to restore reservoir state wettability^17, 18^ Consequently, a sampling strategy that included benign coring fluid and sample preservation coating was pursued to insure that all measurements were performed on native state core material. Fluid contact was limited to native reservoir fluids. Although there is some variation, the pressure capillary data (see Fig. 4, Gao *et al*.^14^) suggest most samples tend to be oil wet. Our two samples were selected to represent a micro-pore and a mixed-pore system, properties are summarized in Table 1. As detailed in previous publications^8, 14^, careful SCAL tests were conducted on these samples using reservoir fluids to establish measures of oil recovery. As these will be the basis for comparison with our models, we show several measures in Table 1. Columns 3 and 4, show the remaining oil (RO) at water breakthrough. In these cases the native state core plug is first saturated with reservoir oil up to residual water saturation. Then formation brine is flooded through the core plug. For RO-USS (unsteady state water flood), this value is obtained by water flooding up to the point of first water appearance at the exit face. For RO-SS (steady state oil/water flood), the steady-state breakthrough value is a calculated property from Johnson-Bossler-Naumann analysis of steady-state relative permeability data^14, 19, 20^. In Column 5, the value for ROS-SS is the end-point saturation for continuous flooding during steady state permeability measurement. This is determined at a fractional oil/water flow of 1/99 as reported by Fullmer *et al*.^8^ with experimental details given in Gao *et al*.^14^.

**Table 1:** Porosity, Microporosity and Remaining Oil Saturation values measured on companion core plugs.

	Porosity^1^	Microporosity, %^2^	RO at Breakthrough USS, PV^3^	RO at Breakthrough SS, PV^4^	ROS Rel.Perm SS, PV^5^
Sample 1	17.4	95	0.34	0.37	0.29
Sample 2	29.3	70	0.61	0.55	0.45

^1^Helium porosimetry.

^2^From TPS analysis^8^.

^3^Remaining oil at water breakthrough for unsteady state water flood, PV = pore volume.

^4^Remaining oil at water breakthrough calculated from steady-state relative permeability data through use of the familiar Johnson‐Bossler‐Naumann (JBN) analysis^14, 19, 20^.

^5^Remaining oil saturation, defined as oil content of core plug after steady-state relative permeability flooding to 1/99 oil/water fraction.

### X-ray Tomography

We used X-ray tomography to characterize the macro-pore size distribution (also see Supplementary Material). CT data were acquired using an X-Tek HMX-160 microCT scanner. This X-ray unit operates with fan-beam geometry and produces broad-spectrum radiation. We utilize a copper filter to remove softer parts of the spectrum. Typical operating conditions are 160 keV and 50 µA impinging on a spot target of 0.5 mm diameter. Samples are scanned at a 30 µm pixel pitch. However, to improve signal to noise, these data are rescaled to 60 µm resolution through use of the ImageJ macro Image/Scale.

The X-Tek vendor software CT-Pro was used to apply beam hardening corrections and for tomographic reconstruction.

### Segmentation and Pore Size Analysis

Unlike conventional XMT analysis, our method uses calibrated X-ray attenuation coefficients measured for each individual voxel^21^. The XMT volumes consist of a 3D matrix of gray scale values representing linear attenuation, I_i_, which we analyze voxel by voxel.

To analyze the pore size distribution, voxels are segmented into three populations: calcite, micro-pore, and macro-pore using two thresholds denoted as, I_pore_, which is the threshold between micro-pores and macro-pores, and I_rock_, which is the threshold between micro-pores and calcite.

After the segmentation, the gray scale value of each voxel, ϕ_n_, is rescaled so that for voxels with I_i_ > I_rock_, ${{\rm{\varphi }}}_{{\rm{n}}}=0$, representing solid rock with zero porosity; for voxels with ${{\rm{I}}}_{{\rm{i}}} < {{\rm{I}}}_{{\rm{pore}}},\,{{\rm{\varphi }}}_{{\rm{n}}}=1$, representing macro-pores with 100% porosity; and for voxels with ${{\rm{I}}}_{{\rm{pore}}}\le {{\rm{I}}}_{{\rm{i}}}\le {{\rm{I}}}_{{\rm{rock}}},\,{{\rm{\varphi }}}_{{\rm{n}}}=({{\rm{I}}}_{{\rm{rock}}}-{{\rm{I}}}_{{\rm{i}}})/({{\rm{I}}}_{{\rm{rock}}}-{{\rm{I}}}_{{\rm{pore}}})$, representing microporosity. The total porosity and the percentage of microporosity of the sample can then be calculated from local porosity of each individual voxel. Specifically, the total porosity, Φ, is calculated using

1 $${\rm{\Phi }}=\frac{{\sum }^{}{\varphi }_{n}}{N}$$

where *N* is the total number of voxels in the sample, and the percentage of microporosity, Φ_s_, is calculated using

2 $${{\rm{\Phi }}}_{S}=1-\frac{{\sum }^{}{\varphi }_{n}\in ({\varphi }_{n}=1)}{N{\rm{\Phi }}}=1-\frac{{N}_{open}}{N{\rm{\Phi }}}$$

where N_open_ is the number of macro-pore voxels with 100% porosity.

The two thresholds, I_pore_, and I_rock_ are selected so that the total porosity and percentage of microporosity are consistent with the values measured by TPS analysis^8^.

After segmentation, the positions and sizes of all open pores are determined. Therefore, we analyze the size distribution of open pores using MATLAB Image Processing Toolbox^22^. Specifically, connected voxels that belong to the “open pore” category are grouped into “clusters”. The volume of each cluster, V_i,open_, is recorded by simply summing up the number of voxels that belong to this cluster. As a measure of size, we calculate the equivalent radius of each open pore cluster, R_i,open_, as

3 $${R}_{i,open}=\sqrt[3]{{V}_{i,open}}/2$$

We then obtain the size distribution of open pores as ${\rm{dV}}/{\rm{dR}}$ vs. R in a semi-log plot shown in Fig. 1. We find the size distribution of open pores in Sample 1 and Sample 2 are well described by the familiar log-normal distribution, *e.g*. Eqn (4), shown as the solid lines in the figure. The fitting parameters are ${a}_{0}=56.45\,\mu m,{s}_{0}=0.68$ for Sample 1 and ${a}_{0}=148.1\,\mu m,{s}_{0}=0.69$ for Sample 2. Because the pore sizes are relatively large compared to the voxel size, we show in the Supplementary Material that the values are not sensitive to our choice of voxel resolution.

**Figure 2:**
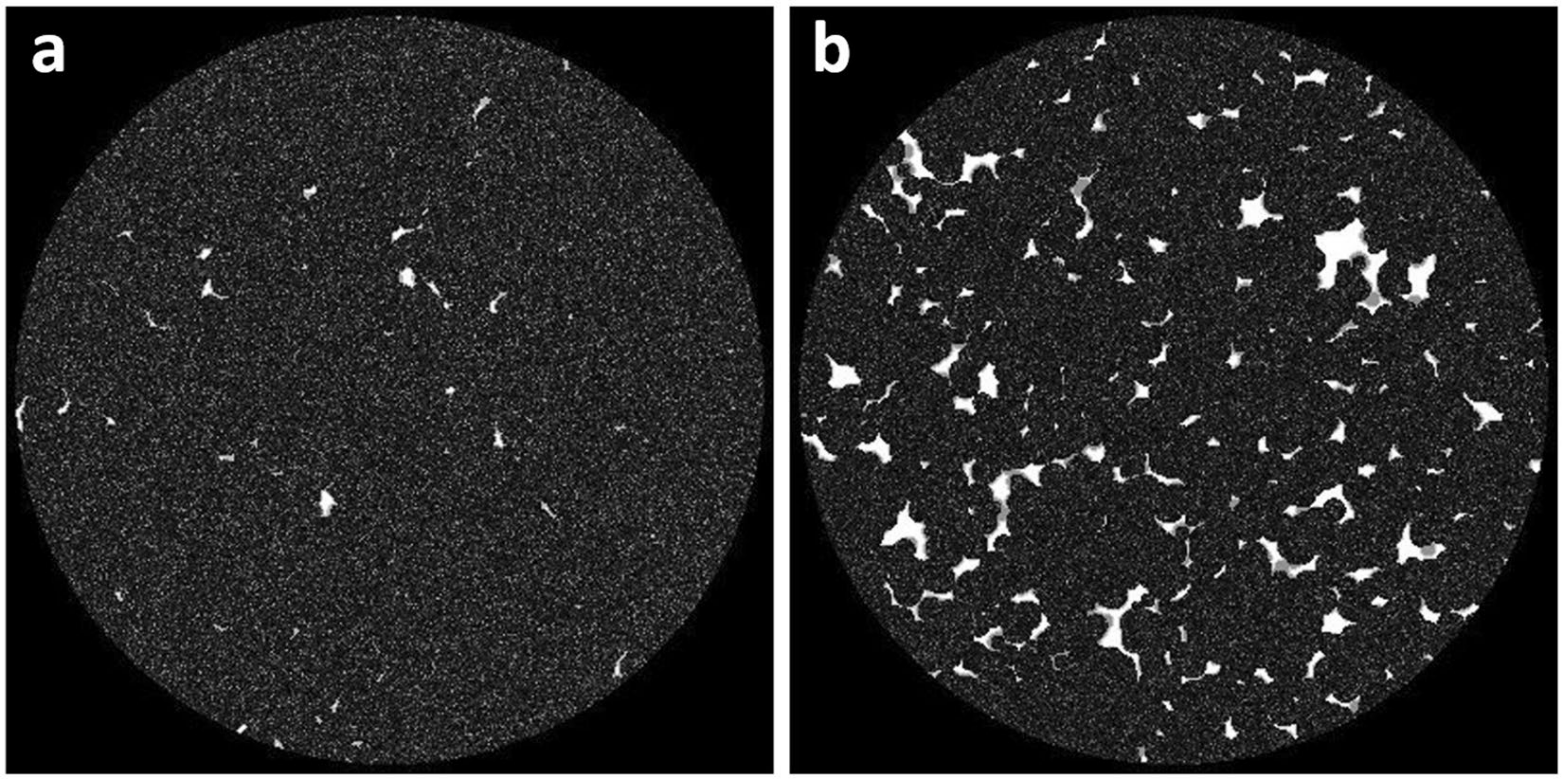
2D slice of sample digital rock with (**a**) 20.6% total porosity and 96% microporosity and (**b**) 25.2% total porosity and 74% microporosity showing a mixture of macro- and micro-pores. This shows good agreement with thin section images from actual carbonate rock samples (see for example Fullmer *et al*.^8^ Figs 4 and 5).

Because the micro-pores are typically < 10 µm, their presence is detected by voxel attenuation ${{\rm{I}}}_{{\rm{pore}}}\le {{\rm{I}}}_{{\rm{i}}}\le {{\rm{I}}}_{{\rm{rock}}}$. From this we obtain quantification of their contribution to the voxel porosity as well as their spatial location (i.e. distribution of similar I_i_ values). However, the size distribution is under constrained. In this Section, we make the assumption of one micro-pore per voxel. Relaxing that constrain, and exploring the sensitivity to micro-pore distributions in latter Sections indicates this to have minimal effect on our conclusions.

### Invasion Percolation Model for Digital Rock

The XMT images of Sample 1 and Sample 2 represent only two examples of carbonate rocks having different percentages of microporosity. To systematize this variation, a digital rock model was created to produce well-defined pore space, and to allow exploration of the variation of macro- to micro-pore.

We use a packing of monodisperse and overlapping spheres to simulate the grainstone. We use a simulation box with a size of 400 × 400 × 600 pixels, and start by randomly placing 8-pixel-radii spheres inside the box. At each step, we compute the volume occupied by the spheres and continue to add spheres until the fraction of sphere-occupied volume reaches a pre-set value. As a result, we generate multiple digital rock models where the volume occupied by spheres represents solid grains, with the remaining volume being interparticle pores. We generated digital rock models with various solid volume fractions, ${{\rm{\varphi }}}_{{\rm{grain}}}$, ranging from 90% to 99%, corresponding to rocks with interparticle pore volume fraction ranging from 10% to 1%. For each solid volume fraction, 10 realizations of that digital model are generated. We determined the void percolation volume fraction through use of the digital flooding algorithm (see below). Our value of ~5% (Fig. S2) is in reasonable agreement with previous literature^15, 16^.

By considering the previously discussed geological mechanism of forming macro-pores and micro-pores in carbonate rocks^9^, the digital rock model interparticle pores will represent the inter-grain, macro porosity in the carbonate rocks. Indeed, the size distribution of inter-particle pores resembles those from the XMT analysis. In Fig. 1, we have excluded digital rocks above the percolation threshold because the inter-particle pores in those rocks form large percolating clusters. It appears that the overlapping sphere model captures the essential character of the macro-pores in the example carbonate rocks.

We next added microporosity to the digital rock models. The distribution of voxel grey scale values, I_i_, suggests that the micro-pores are uniformly distributed throughout the sample, with no evidence of clustering or localization near the grain boundaries. Therefore we incorporate microporosity by randomly assigning gray scale values between 0 and 1 to each voxel belonging to the initially solid grain. We determine the distribution function of those gray scale values from a log-normal distribution of the size of micro-pores.

The volume distribution density function of micro-pores, *f*(*r*), is given by

4 $$f(r)=\frac{1}{\sqrt{2\pi }{s}_{1}r}exp[-{(\frac{\mathrm{log}(r/{a}_{1})}{\sqrt{2}{s}_{1}})}^{2}]$$

where *r* is the radius of a micro-pore, and *a*_1_ and *s*_1_ are parameters for the log-normal distribution.

We make the assumption that each voxel contains one micro-pore, then the local porosity for voxel *n*, ${\varphi }_{n}={(2{r}_{i})}^{3}/{a}^{3}$, where a is the size of each voxel. The volume distribution density of voxels with *ϕ* can be expressed as

5 $$f(\varphi )=\frac{dV(\varphi )}{d\varphi }=f(r)|\varphi \cdot \frac{dr}{d\varphi }=\frac{12}{\sqrt{2\pi }{s}_{1}\varphi }exp[-{(\frac{\mathrm{log}({\varphi }^{\frac{1}{3}}\cdot a/2{a}_{1})}{\sqrt{2}{s}_{1}})}^{2}]$$

The number distribution density of voxels with *ϕ* is then expressed as

6 $$N(\varphi )={N}_{t}f(\varphi )d\varphi =\frac{12{N}_{t}}{\sqrt{2\pi }{s}_{1}\varphi }exp[-{(\frac{\mathrm{log}({\varphi }^{\frac{1}{3}}\cdot a/2{a}_{1})}{\sqrt{2}{s}_{1}})}^{2}]d\varphi $$

where *N*_*t*_ is the total number of voxels inside the solid grain part of the digital model.

There is an additional constraint for the possible distribution of micro-pores. To approximate the porosity of the carbonate rock example, we require the total volume of micro-pores to be 20% of that volume occupied by the grains, *i.e*. ${\varphi }_{micro}=0.2\ast {\varphi }_{grain}$. Therefore, the mean of *ϕ*, will equal 0.2. This constraint determines the possible combinations of *a*_1_ and *s*_1_ for the micro-pore distribution. We select a distribution width, ${s}_{1}=0.25$,which approximates that of the Mercury Intrusion Capillary Pressure (MICP) data. This gives ${a}_{1}=6.5\,\mu m$, corresponding to a mean radius of 6.7 um. It is slightly larger than the average micro-pore size from the example rock, but because it is well separated in size from the macro-pore size, this has no significant effect.

After determining *a*_1_ and *s*_1_, we use a random number generator in MATLAB to generate *N*_*t*_ number of gray scale values between 0 and 1, which follow the required distribution density function, *N*(*ϕ*). These are randomly assigned to voxels that belong to the solid grain part. The 2D slice of resulting digital rock is shown in Fig. 2. The 2D slices of sample digital rock with A) 20.6% total porosity and 96% microporosity and B) 25.2% total porosity and 74% microporosity exhibit a mixture of macro- and micro-pores. These images show good agreement with thin section images from actual carbonate rock samples (see for example Fullmer *et al*.^8^ Figs 4 and 5

**Figure 3:**
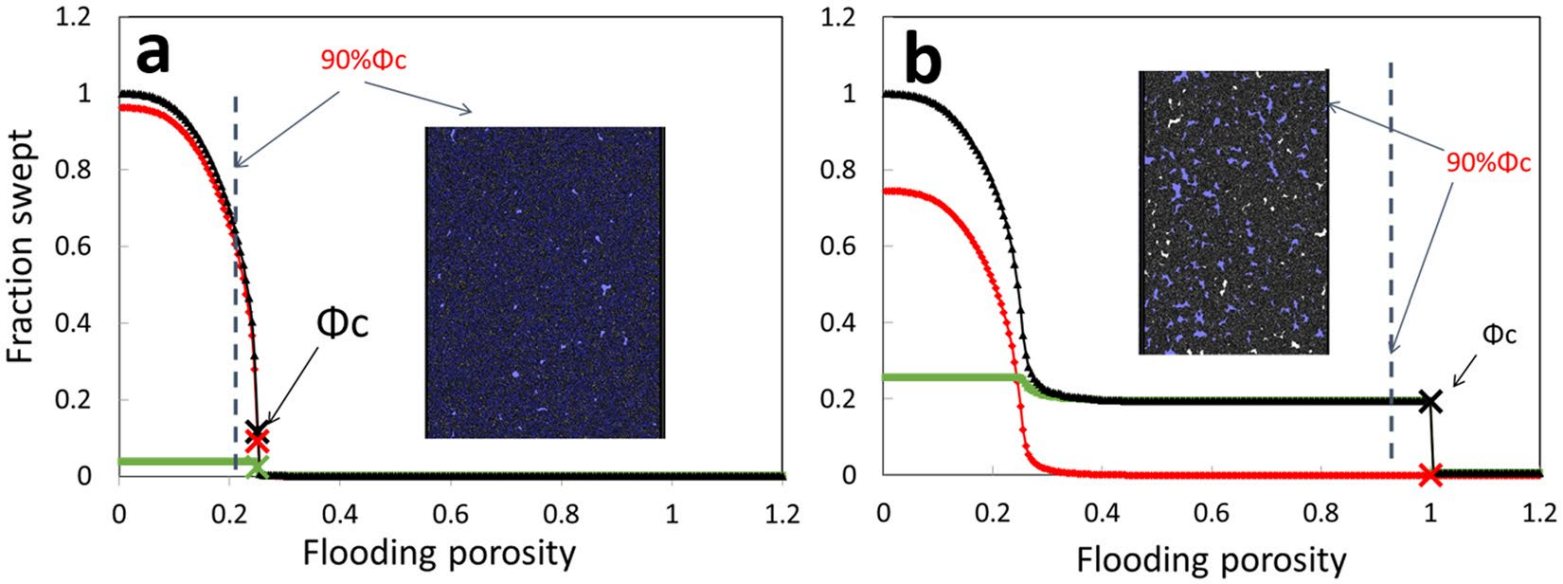
Total cumulative flooded pore volume(black) as a function of the “flooding porosity” for two digital rock models: (**a**): 1% interparticle pores, 96% microporosity and (**b**): 7% interparticle pores, 74% microporosity. Resolved pore and unresolved pore components shown as green and red curves. For (**a**), micro-pores are necessary for percolation (Φ_c_), In the inset, a 2D slice from the 3D volume shows the flooded (blue) volume is uniformly distributed throughout the sample whereas for (**b**), percolation occurs at a flooding porosity equal unity, i.e. open pore percolation, The resulting tenuous connected pathway bypasses the micro-pores. Indicated is our measure of RO, which is flooded volume at 90% Φ_c_.

).

**Figure 4:**
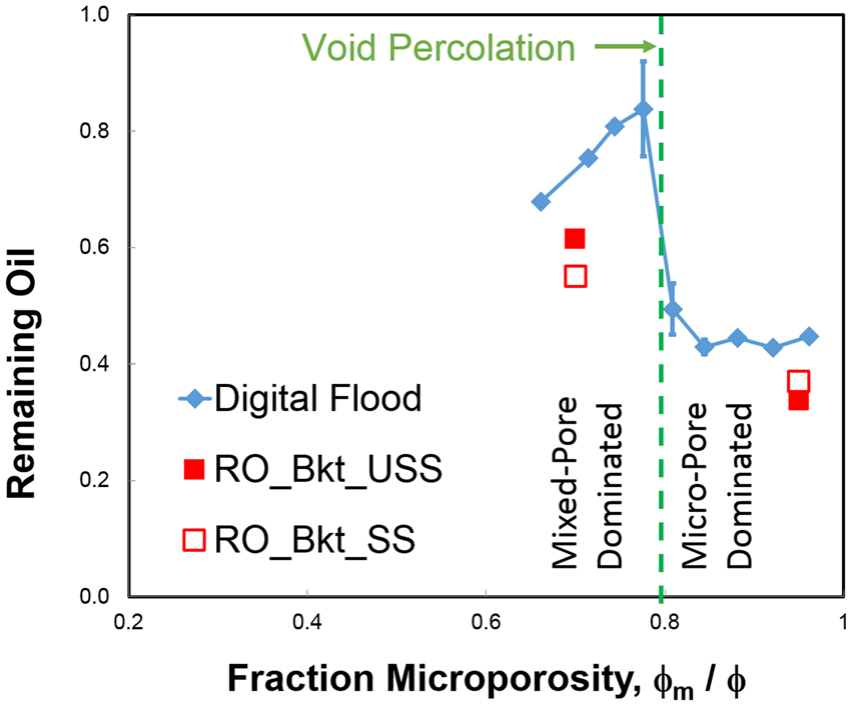
Demonstration of change in remaining oil for digital rock model after crossing over void percolation limit. Jump in magnitude for remaining oil is due to bypass of micro-pores. These results are compared with two experimental measures of remaining oil at water breakthrough: one from unsteady-state flooding (USS) and one derived from steady-state flooding (SS) using Buckley-Leverett analysis.

**Figure 5:**
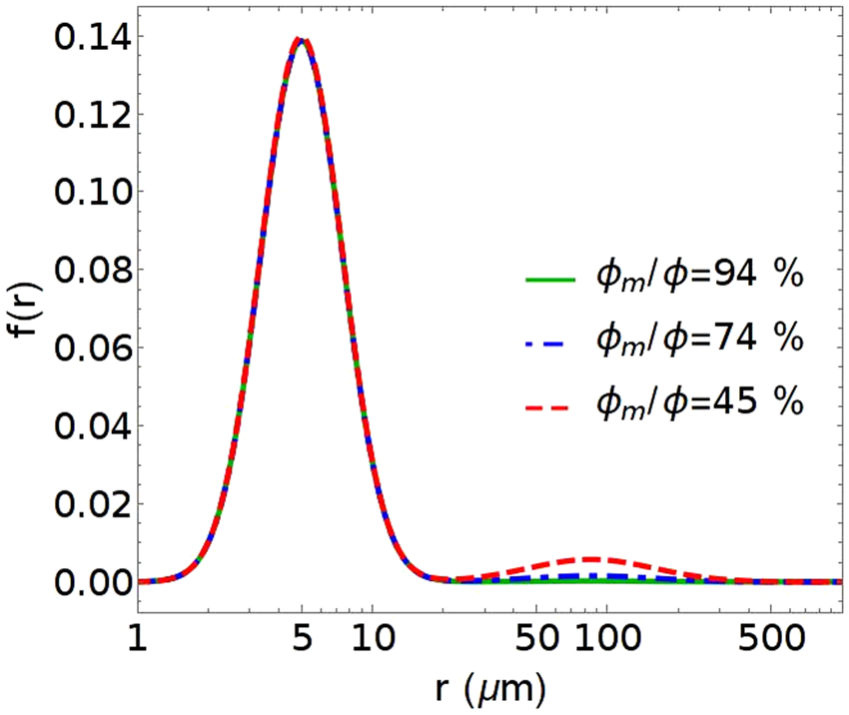
Examples of pore size distributions for the bi-modal pore size function (Equation 9). The microporosity percentage is varied by changing the relative amplitude of each component, leaving the other defining parameters fixed. The following parameters are used: macro-pore a_0_ = 125.3 μm, s_0_ = 0.62 and micro-pore a_1_ = 5.848 μm, s_1_=0.4. Solid green, dashed-blue and dashed red lines correspond to microporosity percentage 94%, 74% and 45% respectively.

### Analyzing Connectivity: Digital Flood

To analyze the connectivity of the pore space in digital rock models, we perform digital flooding using a method introduced in Dunsmuir *et al*.^21^. Specifically, exact Euclidean distances to the nearest surface of the open pore space are calculated for voxels belonging to the “open pore” category, while the voxels that belong to the “microporosity” category keep their local porosity values. In each step, a “flooding porosity”, *R*_*flood*_, is set. Starting from the top slice, all connected voxels with Euclidean distance (open pore) or local porosity (micro-pore) values equal or larger than *R*_*flood*_ are counted towards the “flooded pores”, whose volume is calculated by summing up the pore volume of each flooded voxel. Once the connected network of “flooded pores” reaches the bottom slice, we call it the “breakthrough point”. Afterwards, cumulative flooded pore volume can be plotted as a function of *R*_*flood*_. In Fig. 3 we show those “digital flooding” curves for two digital rock examples, above and below the void percolation limit, 1% and 7% interparticle pores. Clearly, those two digital rocks show very different breakthrough points. For the digital rock with 1% interparticle pores, the breakthrough happens when *R*_*flood*_ is around 0.25, indicating the micro-pores are needed for a percolation network to form. By contrast, the breakthrough *R*_*flood*_ in the digital rock with 7% interparticle pores is exactly 1.0, indicating that open pores alone are enough for a percolation network. Since we have the knowledge on the whether the contribution of flooded pore volume comes from the open pore or micro-pore, we can further decouple the curves into the resolved pore and unresolved pore parts, shown as green and red curves in Fig. 3. Similar results are obtained for the XMT volumes from actual rock Samples 1 and 2. Specifically, for Sample 1 which has 95% microporosity, the breakthrough *R*_*flood*_ ~0.5 while that for Sample 2 with 70% microporosity *R*_*flood*_ = 1.0.

We now use these results to investigate how void fraction affects remaining oil for an invasion percolation model. This digital flood simulates the volume invaded by a non-wetting fluid (consistent with the oil-wet nature of these rocks), and we equate the swept pore volume with the produced oil from an initially oil-saturated rock. To simulate an end point for the flooding and calculate remaining oil, we take the breakthrough as an important marker. At that point in an actual immiscible flood experiment, the high-efficiency 1:1 volume displacement ends. After that, oil production with further flooding exhibits an asymptotic approach to a final saturation state, often described by the familiar Johnson-Bossler-Naumann (JBN) analysis^14, 20^. We approximate remaining oil for the digital flooding by selecting *R*_*flood*_ equal to 90% of the *R*_*flood*_ at the breakthrough point. Unlike the remaining oil exactly at breakthrough, which is highly sensitive to the geometry of an individual digital rock realization and not reproducible, this remaining oil value is robust for all individual realizations. We take the average over 10 realizations of the model at each void fraction and these values along with ± one standard deviation are plotted in Fig. 4.

Fullmer *et al*.^8^ had observed that above 80% microporosity there is a significant decline in permeability with an accompanying improvement in oil recovery. These results suggest this is due to the void percolation limit. This is denoted by the green line in Fig. 4. Void percolation is rather insensitive to details of the grain shape, or polydispersity^15, 16^. Thus, we can expect this to be a common feature for grainstones having a void content above approximately 5 percent. However, the value of microporosity percentage is not uniquely defined because grains themselves contribute to the total porosity. We quantify the relationship between void percolation and microporosity in the Supplementary Material. With void percolation at 0.05 volume fraction, our assumption of grains having 20% microporosity gives the transition here at 79% microporosity, i.e. ${\varphi }_{m}/\varphi =(1-0.05)$ $\ast 0.2/(0.05+(1-0.05)\ast 0.2)$.

To compare with experimental results we plot the two values of RO at breakthrough from Table 1. At high microporosity, there is remarkably good agreement between the digital flooding values and both experimental values. Here the USS and SS values are in good agreement because the assumption of a uniform sweep, inherent in the JBN analysis, is a good approximation. For the mixed-pore sample there is less agreement between experimental values, undoubtedly due to flow fingering for the USS experiment. Invasion percolation captures the physics of this fingering flow and our digital flooding results are in closer agreement with this data point.

Although this invasion percolation model provides a plausible explanation for increased sweep efficiency above 80% microporosity, it is unsuitable to simulate the mixed-water/oil fluid flow regime above breakthrough. There, one will observe an asymptotic approach to final residual oil, and it is those ROS values which are reported by Fuller *et al*. to be directly related to microporosity percentage. For that reason, we need another model, and in the next section we utilize one based on effective medium theory.

### Effective Medium Model

In this part of the paper, we consider a pore geometry derived from a random spatial distribution of micro-pores and macro-pores and apply an effective medium model^23–25^ to predict the ROS in these heterogeneous carbonate samples. The effective medium model is one of the most successful analytical treatments of the transport properties of composite materials due to its mathematical and conceptual simplicity^25^. It has been demonstrated that the predictions of effective medium model provides reasonable estimates of the effective transport properties and are in excellent agreement with many observations in the literature^25–27^. In our particular case, the validity of the effective medium model is justified by the random distribution of micro-pores and macro-pores as is evident in examining the pore geometry of actual grainstones as well as our digital rock model (see Fig. 2). More importantly, our predicted ROS values agree very well with the experimental measurement without involving any arbitrary fitting parameters, which further validates our effective medium approach. In addition, the sensitivity analysis, as shown in Fig. 7

**Figure 6:**
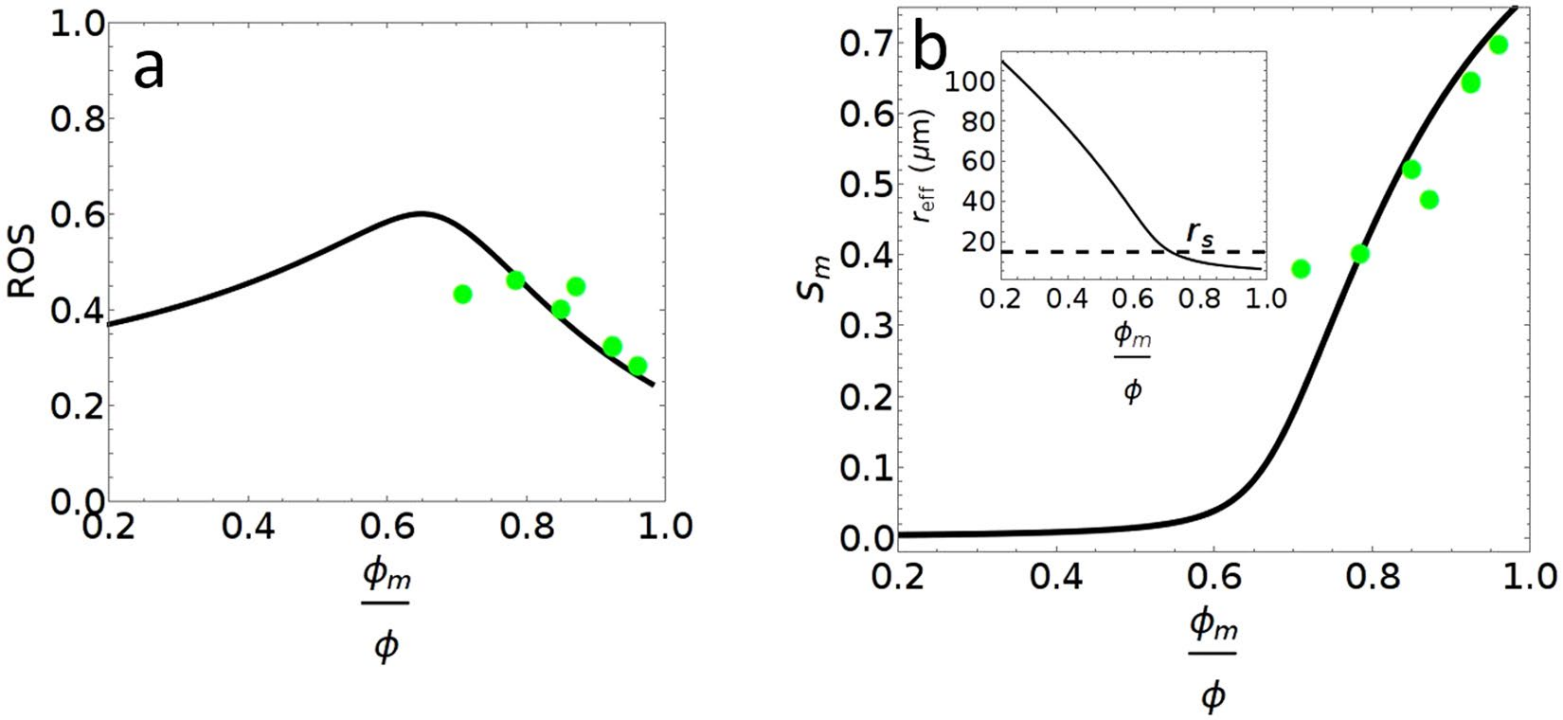
(**a**) Remaining oil saturation (ROS) versus micro-pore fraction ϕ_m_/ϕ. (**b**) Micro-pore sweep efficiency (*S*_m_) vs. micro-pore fraction Black lines are the model predictions. The following parameters are used: Macro-pore a_0_ = 125.3 μm, s_0_ = 0.62 and micro-pore a_1_ = 5.848 μm, s_1_ = 0.4. Green dots are experimental waterflood data from Fullmer *et al*.^8^ Figure 15.

, demonstrates the robustness of our effective medium model. Finally, in the Supplementary Materials, we show that there is a simple mapping between the digital rock model and the effective medium model based on pore size distribution by comparing the micro-pore volume to macro-pore volume ratios.

#### A. Effective Medium Theory

For any random, heterogeneous system, the effective hydraulic conductivity can be calculated using effective medium theory by solving the following equation:

7 $${\int }^{}d\sigma P(\sigma )\frac{\sigma -{\sigma }_{eff}}{\sigma +2{\sigma }_{eff}}=0$$

where *P*(σ) is the probability for the local value of σ, the hydraulic conductivity^28^.

Using the relation between hydraulic conductivity σ and pore size σ ∝ r^2^, Equation (7) can be rewritten as:

8 $${\int }^{}drf(r)\frac{{r}^{2}-{r}_{eff}^{2}}{{r}^{2}+2{r}_{eff}^{2}}=0$$

where the effective pore size *r*_eff_ is related to effective conductivity σ_eff_ of the system.

To obtain the remaining oil saturation, we propose that oil in pores with sizes larger than r_eff_ is completely displaced, 100% sweep. It then follows that the fraction of oil that can be displaced in pores with size r < r_eff_ is proportional to the velocity ratio $v(r)/v({r}_{eff})={r}^{2}/{r}_{eff}^{2}$, with *v*(*r*) being the flow velocity in pore size *r*. Thus, we see that, in contrast to our void percolation model, here pores of all sizes contribute to the recovered oil.

#### B. Bimodal Pore Size Distribution Function

We now consider the impact of a specific bi-modal pore size distribution denoted as *f*(*r*). We use a log-normal distribution to describe the pore size distribution^29^, and note that other distributions will produce similar results:

9 $$f(r)=\frac{{w}_{s}}{\sqrt{2\pi }{s}_{1}r}exp[-{(\frac{\mathrm{log}(r/{a}_{1})}{\sqrt{2}{s}_{1}})}^{2}]+\frac{1-{w}_{s}}{\sqrt{2\pi }{s}_{0}r}exp[-{(\frac{\mathrm{log}(r/{a}_{0})}{\sqrt{2}{s}_{0}})}^{2}]$$

with *w*_s_ being the volume fraction of micro pores; *a*_1_ and *a*_0_ are radii for the micro and macro pores, respectively; *s*_1_ and *s*_0_ are the corresponding standard deviations of micro and macro pore size distributions.

This distribution satisfies the normalization condition,

10 $${\int }_{{r}_{min}}^{\infty }f(r)dr=1$$

This condition ensures that the volume fractions in this Section are all normalized to the total porosity and are reported as a fraction of the pore volume. Void percolation, which is defined as a void fraction relative to the sample volume cannot be directly analyzed here.

To calculate the volume fraction of macro-pores and micro-pores we introduce a lower bound cutoff for the pore size distribution, r_min_, and a length scale separating the macro and micro pore size denoting as r_s_. Therefore, the volume fraction of micro-pores is given by

11 $$\frac{{\varphi }_{m}}{\varphi }=\frac{{\int }_{{r}_{min}}^{{r}_{s}}f(r)dr}{{\int }_{{r}_{min}}^{\infty }f(r)dr}\approx {w}_{s}$$

where for the example shown *r*_min_ ~4 nm. The choice of *r*_min_ does not affect the final results as long as *r*_min_ $\ll \,$ *a*_1._

To make connection with our rock model, the length scale for our f(r) is r_s_ = 15 μm. Note that this cutoff is only needed to calculate the microporosity percentage, and should not be confused with r_eff_, which is the key value that determines sweep characteristics for the pores. The final results are insensitive to the choice of r_s_ as long as f(r_s_) is close to zero at that point or equivalently saying that the micro and macro pore sizes are well separated, which is indeed true for the rock samples of our interests. Under this condition, it can be shown that ${\varphi }_{m}/\varphi ={w}_{s}$.

The parameters for the *f*(*r*) used in our example were obtained by considering the results from the digital rock pore structure for macro pores combined with MICP data for 100% Type I micro-pore rocks. The following parameters are used in our calculations: Macro-pore a_0_ = 125.3 μm, s_0_ = 0.62 and micro-pore a_1_ = 5.848 μm, s_1_ = 0.4.

#### C. Remaining Oil Saturation

The procedure for determining ROS goes as follows. We first utilize the pore size distribution, equation (9), along with the constraint imposed by equation (8) to solve for r_eff_, which has a unique value for a given pore size distribution. It can be larger or smaller than r_s_ depending on the microporosity percentage.

Having determined r_eff_, we then solve for the remaining oil saturation (ROS) using:

12 $$ROS={\int }_{{r}_{min}}^{{r}_{eff}}f(r)(1-\frac{{r}^{2}}{{r}_{eff}^{2}})dr$$

The following equation can then be used to calculate the micro-pore sweep efficiency, which is a measure of how much oil from micro-pores contributes to produced oil.

13 $${S}_{m}=1-\frac{{\int }_{{r}_{min}}^{{r}_{eff}}f(r)(1-\frac{{r}^{2}}{{r}_{eff}^{2}})dr}{{\int }_{{r}_{min}}^{{r}_{s}}f(r)dr}$$

These functions are plotted in Fig. 6 and compared to experimental data. The ROS values are those from Fullmer *et al*.^8^ Figure 15. We calculate the *S*_*m*_ values from that same data as follows. Making the assumption that the volume of produced oil (1-ROS) consists of fully swept macro-pores and partly-swept micro-pores, we take the difference in volumes and normalize them to obtain ${{\rm{S}}}_{{\rm{m}}}=[(1-{\rm{ROS}})-(1-{{\rm{\Phi }}}_{{\rm{s}}})]/{{\rm{\Phi }}}_{{\rm{s}}}$, where Φ_s_ is the microporosity from TPS analysis.

**Figure 7:**
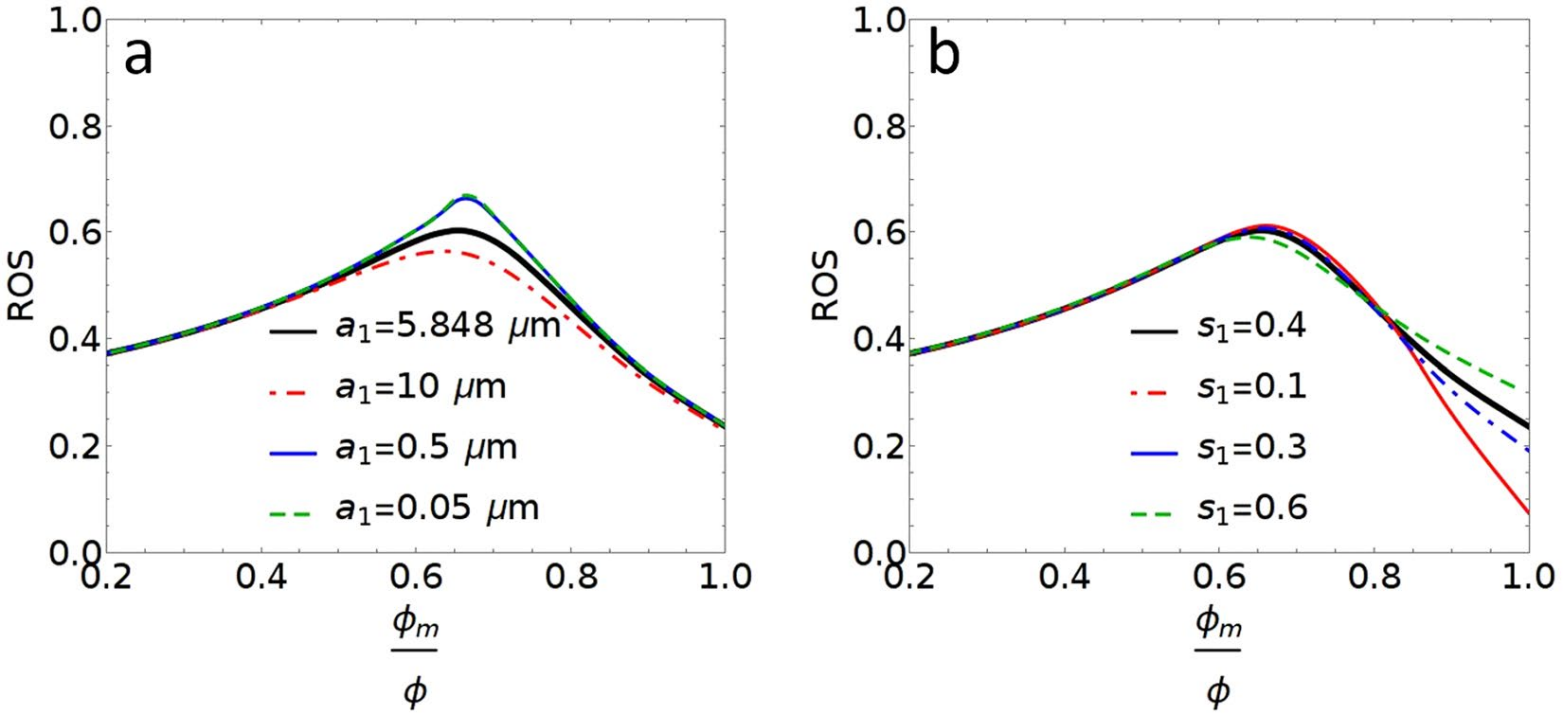
(**a**) Demonstration of ROS variation for different micro-pore radius. For all curves the following model parameters are fixed s_1_ = 0.4, macro-pore ${s}_{0}=0.61728\,\text{and}\,{a}_{0}=125.304\,\mu m$. Black, red, blue and green lines are for respectively a_1_ = 5.848 μm, 10 μm, 0.5 μm and 0.05 μm; (**b**) Demonstration of ROS variation with changes in the standard deviations of the micro-pore distribution. For all curves the following model parameters are fixed, a_1_ = 5.848 μm, macro-pore ${s}_{0}=0.61728\,\text{and}\,{a}_{0}=125.304\,\mu m$ Black, red, blue and green lines are for s_1_ = 0.4, 0.1, 0.3, and 0.6.

The ROS function in equation (12) provides an excellent description of the observed dependence over the range of experimental microporosity. The *S*_*m*_ function (equation (13)) similarly closely matches the experimental data. It is remarkable that the only input needed for this calculation is the pore size distribution. Recall that the constraining equation (equation (8)) uniquely gives a r_eff_ value upon specification of f(r). This is the only parameter necessary to obtain ROS. As there is no void percolation limit explicitly considered here, there is no abrupt transition in ROS at 80% microporosity. However, there is clearly an increase in ROS with decreasing microporosity, reaching a maximum at ϕ_m_/ϕ ~ 0.65. Examining Fig. 6b, we find that this ROS increase is associated with an important change in the swept volume, with S_m_ reaching a minimum, i.e. micro-pores contributing little oil. Also, looking at the inset of that figure we see that this is where r_eff_ > r_s_. Recall that for pores with r > r_eff_ these pores are fully swept. Hence at this microporosity content, the produced oil will mostly come only from macro-pores. Therefore, although the transition is not so sharply defined as for the invasion percolation model, it leads to a similar condition as that for our void percolation model.

With further reduction in micro-pore content (macro-pore increase), ROS is predicted to show a decrease. This is expected because the pores are now dominated by macro pores and the system becomes more homogeneous.

#### D. Sensitivity Analysis

Fullmer *et al*.^8^ show in their Fig. 3 that the character of microporosity can vary across different carbonates. This variation changes the average pore size as well as the breadth of the distribution. In this section, we explore how these changes in the pore size distributions affect the model predictions. Through these results, we gain an understanding of the robustness of the conclusions to changes in pore size distribution.

There are several notable conclusions can be drawn from these sensitivity analyses: First, the overall shape of the ROS versus microporosity curve is preserved throughout these alterations, which suggests that the response to changing micro-pore content will be similar for most grainstones. This trend is supported by the experimental data on minimum oil saturation versus percent microporosity from a broad sample suite, Fullmer *et al*.^8^ Fig. 7(b). Second, the effective medium model predicts poorer ROS for a mixed pore rock when the micro-pores are very small. The model also predicts better ROS when the micro-pores are more uniform in size.

## Conclusions

This work has demonstrated how we can create a digital rock model which exemplifies the key structural elements of the carbonate grainstone examples. The mechanism employed closely replicates the inter-grain and intra-grain pore types resulting from carbonate diagenesis. This generalization of the rock geometry allows exploration of a wider range of rock types than can be easily obtained from the field.

We explore the oil recovery from this model rock using two approaches. Recognizing that void percolation can be an important feature, we utilize an invasion percolation model to demonstrate how variation in microporosity content can drive a significant improvement in recovery efficiency above about 80% microporosity, as is observed experimentally by Fullmer *et al*.^8^. While this model can predict a recovery factor increase at immiscible fluid flooding breakthrough, it cannot predict the ultimate recovery. For this, we utilize an effective medium model, showing that the recovery versus microporosity percentage is in quantitative agreement with experiments. This analysis shows that oil is produced from both micro- and macro-pores for the higher microporosity percentages typical of low ROS carbonate grainstones. As the microporosity percentage declines, the transition to poor ROS occurs more gradually than for the invasion percolation model. Significant micro-pore bypass and maximum ROS occurs close to ~65% microporosity. These results also suggest that an accurate analysis of the pore size distribution is sufficient to predict recovery factors. From analysis of the sensitivity of recovery to pore size distribution, we conclude that improved recovery can be realized if one can identify rocks having a narrow pore size distribution of micropores. As pointed out by Kaczmarek *et al*.^9^ there seem to be three characteristic petrophysical microporosity types (I, II, III), respectively exhibiting reduced crystal sizes and smaller pore-throat radii. Our sensitivity analysis allows a prediction on how these, combined with a macro-pores will affect oil recovery.

## Electronic supplementary material

Supplementary Information
