# Supplementary material for: Modeling Oil Recovery for Mixed Macro- and Micro-Pore Carbonate Grainstones
Source: Sci Rep. 2017 Aug 29;7:9780. doi: 10.1038/s41598-017-09507-4 (PMC5575129; doi:10.1038/s41598-017-09507-4)
Supplement: Supplementary file 1 — Supplementary Information [file 41598_2017_9507_MOESM1_ESM.docx]

Modeling Oil Recovery for Mixed Macro- and Micro-Pore Carbonate Grainstones

Supplement

Ye Xu, Qiuzi Li, Hubert King

Corporate Strategic Research

ExxonMobil Research and Engineering Co.

Annandale, NJ 08801

## A. Effect Of XMT Resolution On Measured Open-Pore Size Distribution

As discussed above, the samples are scanned at a 30µm pixel pitch but rescaled to 60µm to improve signal to noise. To test the effect of voxel size on the analyzed macro-pore size distribution, we performed the analysis from section: **Segmentation and Pore Size Analysis** on the 30µm pixel data. The results for the two resolutions are compared in Fig. S1. Not surprisingly, the 30µm pixel results are shifted to slightly smaller sizes. However, the distribution of sizes remains about the same, as shown by similar widths for the two LogNormal fits. The peak of the distribution is shifted by approximately 10µm. Therefore, the use of reduced resolution XMT images does not have a significant impact on the parameters we use for the digital rock model.

| 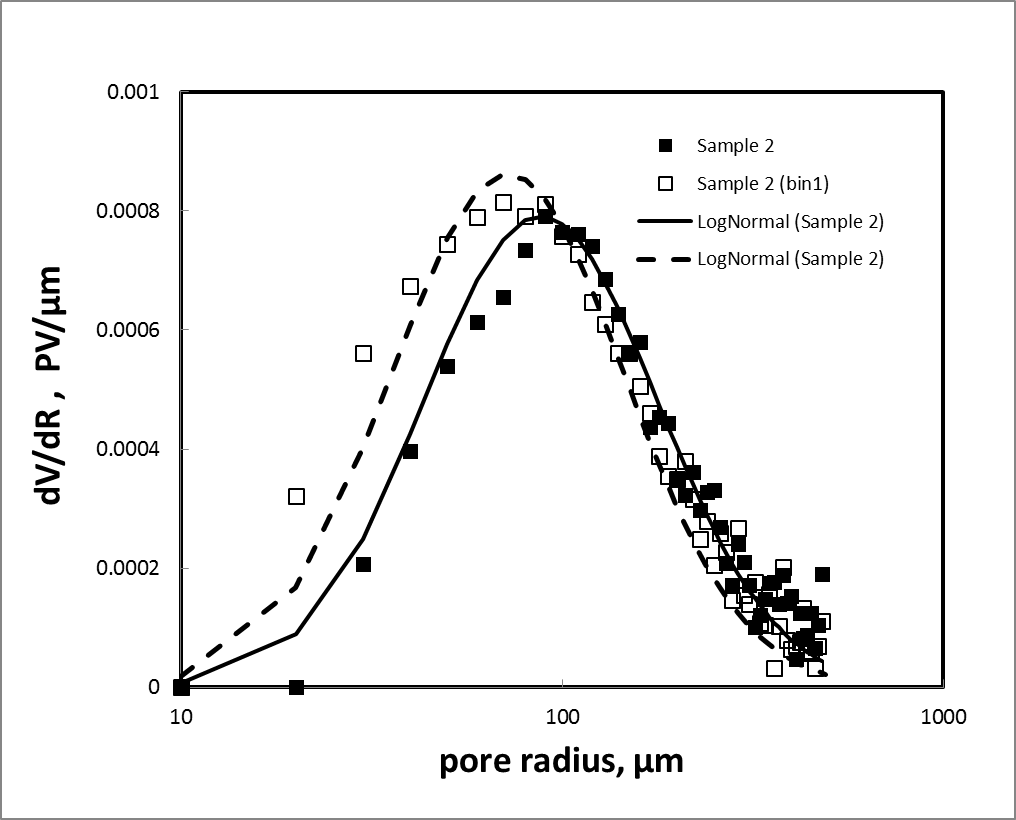 |
| --- |
| Figure S1. Macro-pore size distribution analyzed using original XMT image (open square) and reduced-resolution XMT image (filled square) of Sample 2. The solid and dashed lines are log-normal fits to those two datasets. |

## B. Percolating Threshold For Intergrain Voids

With increasing volume fraction of intergrain voids in the digital rock model, those voids will start to percolate. As discussed in the main text, we run multiple (10) realizations at each void volume fraction. For each, we test whether there is a cluster of voids that span from top to the bottom for the simulation box. We count the number of such percolating realizations and ratio that to the number of realizations, 10. This gives the probability of percolating vs. void volume fraction, plotted in Fig. S2. For our simulations, voids start to percolate when the volume fraction reaches ~5%. This result is consistent with earlier work which found that the percolation thresholds for inter-sphere voids is 3-5%. [^1^](#_ENREF_1)^,^[^2^](#_ENREF_2)

|  |
| --- |
| Figure S2. Probability of percolating vs. void volume fraction in digital rock models. |

## C. Digital Rock Model Constraints

The following equations govern the porosity evolution of the digital rock model in the accompanying paper. There are micropores V_μ_ and macropores V_v_. As detailed in the accompanying paper, these are related through the generation process. The micropores occupy the solid space not occupied by macropores, by a fixed fraction of that space, 0≤y≤1. In the accompanying paper y=0.2. The space occupied by the macro pores is the inter-sphere space generated by the process of addition of interpenetrating spheres to a fixed system volume. The void fraction is fixed by halting sphere addition and is defined through an input variable, V_v_ =xV. The quantity V is the system volume and 0≤x≤0.38 (limited by RCP). By construction, the total porosity increases as the fraction of voids increases, matching the behavior of the grainstones. However, the upper bound of x is governed by the need to create a space-filling grain pack, hence maximum x=0.38.

The following equations relate important quantities to x and y of the model.

$$V_{\mu}=y\left( V-V_{v} \right)=V(y-xy)$$

$$\Phi_{\mu}=\frac{y(V-V_{v})}{xV+y(V-V_{v})}=\left[ \frac{x}{y-xy}+1 \right]^{-1}$$

$$\phi=\frac{xV+y(V-V_{v})}{V}=x-xy+y$$

$$\frac{V_{\mu}}{V_{v}}=\frac{y(V-V_{v})}{V_{v}}=\frac{y-xy}{x}$$

As we explain in the accompanying paper, this model was selected for grainstones because it seems to capture their essential geometry and replicates observed textures. The one free parameter, y, is constrained by the measured porosity for our nearly 100 % microporous sample, giving y=0.2. For these conditions, an important transition in transport occurs at 80% microporosity. For values above this, the system is below the void percolation limit and transport is governed by the micropore network. The percolation limit for our digital rock model occurs at x~0.05.

Percent microporosity, $\Phi_{\mu}$, is only a partial description of the pore space, evident through the second equation above which requires both x and y. Therefore the void percolation transition will occur at various percent microporosity values depending on the choice for y. This is important because Fullmer at al. [^3^](#_ENREF_3)discuss the tendency for different grain types to exhibit different levels of microporosity, for example due to different composition or internal geometry. With our digital model we can explore how the void percolation transition will shift for various values of y, shown in figure below.

|  |
| --- |
| Figure S3. Line shows calculated void percolation limit. To right of line the flow is dominated by micropores and to left mixed pore flow occurs. For their grainstones, Fullmer et al. observe a significant decrease in permeability when microporosity exceeds 80%. This figure shows how this transition will change for grains exhibiting different degrees of micro pore conversion. |

## D. Digital Rock Model With Diagenesis

Aside from a simple variation of the degree of conversion for the grains, other grainstones can be constructed. Consider three diagenetic processes that could drive void disappearance: simple compaction, cementation, and combined compaction and grain recrystallization. The overlapping sphere model is a good description for the first scenario, we now construct models for the other two cases and compare all three.

With the addition of a parameter describing the cement content, zV, with 0≤z≤0.38, the evolution of the macro pores from their initial volume $V_{v}^{0}$ can be written as $V_{v}$=$V_{v}^{0}$ - zV and the pore space evolution with z described as follows:

$$V_{\mu}=y\left( V-V_{v}^{0} \right)$$

$$\Phi_{\mu}=\frac{y\left( V-V_{v}^{0} \right)}{{(V}_{v}^{0}-zV)+y\left( V-V_{v}^{0} \right)}$$

$$\phi=\frac{{(V}_{v}^{0}-zV)+y\left( V-V_{v}^{0} \right)}{V}$$

$$\frac{V_{\mu}}{V_{v}}=\frac{y\left( V-V_{v}^{0} \right)}{{(V}_{v}^{0}-zV)}$$

As the fraction of cement increases, the rock will eventually reach the point where it is fully cemented and zV=$V_{v}^{0}$. We write z as a total fraction of the rock volume to make it easier to relate to an experimental measurement, for example the fraction cement in the total area of a thin section area. In this formulation, one needs an initial estimate of the inter-grain porosity. One possibility is an estimate from grain packing. As the cement content increases and the void content approaches zero, the micro pores may become isolated from one another and fail to create a flow path. This is difficult to quantify, and we neglect that effect in the plots below.

In the second model, we allow interpenetrating spheres (x→0) but combine this with a variation in y such that the resulting porosity is constant. For diagenesis, this would amount to increased recrystallization accompanying compaction. We utilize the first set of equations, applying a new set of constraints for x and y for the overlapping sphere model.

We now compare all three models:

|  |
| --- |
| Figure S4. In (a) the void volume fraction declines as microporosity approaches 100%. The horizontal line indicates the percolation limit for void percolation, at values below that line transport will be micropore dominated. This transition occurs close to 80% for all three models. In (b) we plot total porosity. When the recrystallization of grains is fixed, (OverlapSphere and CementSphere) the increase in microporosity is accompanied by a porosity loss. The porosity can be conserved if the recrystallization of the grains increases as the void fraction declines (FixPhi). The grain recrystallization increases from y=0.1 to 0.2 across the plotted range. |

## E. Generalized Model for Oil Recovery

The impact on oil recovery from the variations just described, can be investigated through use of the effective medium model. Each scenario above changes the proportion of micro and macro porosity. If we make the additional assumption that the pore size distributions of the macro and micro pores remain unchanged, then we can map recovery onto percent microporosity through comparing with effective medium calculations.

Consider the pore size for the effective medium model, Eqn. 9 in the accompanying paper. Through equations 10 and 11, we see that a unique identity for a given model is obtained by the ratio of micro to macro pores, i.e w_s_/(1-w_s_). We calculate that parameter for each model and compare with the effective medium result, discovering that all models map to an equivalent ratio as that from the effective medium model. Hence, the oil recovery versus percent microporosity for the three scenarios considered will follow that for the effective medium model. See Figure 6 of the accompanying paper. To emphasize the equivalence, we plot here the micro/macro ratio for all models

|  |
| --- |
| Figure S5. The ratio of micro-to-macro pore volume for all scenarios considered here follow an identical trend and therefore the oil recovery for each should be the same as that calculated for the effective medium theory. This presumes that only the proportion of each pore type changes, leaving the pore size distribution within each component unchanged. See the accompanying paper for calculations on how change in pore size distribution will alter recovery. |

The surprising finding here is that for the variety of diagenesis types considered, all of which alter the macro pore content differently, the scaling of oil recovery with percent microporosity continues to be a valid approach.

1 Van der Marck, S. Network approach to void percolation in a pack of unequal spheres. *Physical Review Letters* **77**, 1785 (1996).

2 Yi, Y. Void percolation and conduction of overlapping ellipsoids. *Physical Review E* **74**, 031112 (2006).

3 Fullmer, S. M. *et al.* in *IPTC 2014: International Petroleum Technology Conference* Vol. 17629 (International Petroleum Technology Conference, Doha, Qatar, 2014).
